# Management of Hypertension Using a Plant-Based Diet Among Farmers: Protocol for a Mixed Methods Study

**DOI:** 10.2196/41146

**Published:** 2023-04-03

**Authors:** Tantut Susanto, Hanny Rasny, Fahruddin Kurdi, Rismawan Adi Yunanto, Ira Rahmawati

**Affiliations:** 1 Department of Community, Family, and Geriatric Nursing Faculty of Nursing Universitas Jember Jember Indonesia; 2 Department of Emergency and Critical Care Nursing Faculty of Nursing Universitas Jember Jember Indonesia

**Keywords:** hypertension, farming, plant-based diet, nursing

## Abstract

**Background:**

Farmers in Indonesia have a high risk for hypertension owing to their lifestyle and working environment. Diet management is a solution to reduce hypertension, and Indonesia has natural resources in the agricultural sector that could help manage hypertension. Optimizing vegetable and fruit intake in a plant-based diet (PBD) could help maintain blood pressure among farmers in Indonesia.

**Objective:**

This study aims to explore the health problem of hypertension and the characteristics of local food sources to formulate a PBD menu for treating hypertension, as well as assess the prevalence of hypertension, level of acceptability of a PBD, and associated sociodemographic factors. Further, we want to examine the effectiveness of a community-based nursing program for managing hypertension using a PBD.

**Methods:**

We will use the exploratory sequential mixed methods approach. There will be a qualitative study (phase I) in 2022 and a quantitative study (phase II) in 2023. We will analyze data using a thematic framework in phase I. In phase II, the study will involve (1) questionnaire development and validation; (2) examination of the prevalence of hypertension, the level of acceptability of a PBD, and the associated factors; and (3) a randomized controlled trial. We will recruit farmers with hypertension who meet the study criteria. Moreover, in phase II, we will invite expert nurses and nutritionists to assess the face and content validity of the questionnaire. We will use multiple logistic regression models to estimate the associated sociodemographic factors and the level of acceptability of a PBD. Furthermore, a linear generalized estimating equation will be used to estimate the parameters of a generalized linear model with a possible unmeasured correlation between observations from different time points for systolic and diastolic blood pressure.

**Results:**

A model PBD for hypertension management is expected to be developed. In 2022, we will collect information on hypertension and the characteristics of local food sources for managing hypertension, and will formulate a PBD menu to treat hypertension among farmers. In 2023, we will develop a questionnaire to assess the acceptability of a PBD to manage hypertension among farmers, the prevalence of hypertension, and the sociodemographic factors associated with hypertension among farmers. We will implement a community-based nursing program for managing hypertension using a PBD among farmers.

**Conclusions:**

The PBD model will not be readily available for other agricultural areas since validation of local food variation is required to design the menu. We expect contributions from the local government to implement the intervention as one of the policies in the management of hypertension for farmers in the agricultural plantation areas of Jember. This program may also be implemented in other agricultural countries with similar problems, so that hypertension can be optimally treated among farmers.

**International Registered Report Identifier (IRRID):**

PRR1-10.2196/41146

## Introduction

Hypertension contributes to 12.8% of the total 7.5 million deaths worldwide [1]. The prevalence of hypertension in Indonesia is 33.4% [2], while in East Java province, it is 20.43% [3]. Farming communities have a high risk of health problems [4], including hypertension [5-7]. Hypertension among farmers in Indonesia has reached 36.1% [8]. Our previous study found that the incidence of hypertension among farmers in agricultural areas was 35.8% [9]. This shows the magnitude of the problem of hypertension among farmers, and there is a need for the management of care using an agricultural health nursing (agronursing) approach to realize the health status of farmers.

Agronursing can facilitate occupational health and safety among farmers [9]. Farmers’ health problems are generally caused by a lack of knowledge and skills in health management, which can lead to hypertension [10]. Most farmers with hypertension have a low level of education, so skills in self-care management are lacking [11]. Hypertension among farmers is also caused by an unhealthy lifestyle [9] and inadequate nutrition [12]. High consumption of unhealthy foods among farmers, such as high-sodium foods [6], and low knowledge about diet [13] can increase the risk of hypertension in farmers. Therefore, dietary management with a plant-based diet (PBD) needs to be introduced, and the basic principles of hypertension nursing care for farmers need to be developed.

Natural agricultural resources should be considered in the management of hypertension among farmers. The basic principles of hypertension care among farmers should be developed through optimizing the agricultural sector’s potential by consuming a PBD that prioritizes vegetables and fruits [14]. Therefore, it is necessary to formulate the basic concepts and principles of a PBD in nursing care management for farmers with hypertension based on the agronursing approach.

The agronursing approach to treat hypertension among farmers focuses on promotive and preventive measures to reduce the risk of disease occurrence or disease worsening from previous conditions [15]. Moreover, it focuses on the health status of individuals in carrying out their work (in this case, farmers who work in fields) in order to improve overall health (physically, mentally, and socially) [16]. The PBD concept is expected to be able to overcome the problem of hypertension among farmers in agricultural areas, which is synergistic with the culture and local wisdom of the agricultural population. Therefore, the objectives of this study are as follows: (1) to explore the health problem of hypertension among farmers and the characteristics of local food sources of vegetables and fruits that can be used in a PBD to manage hypertension; (2) to examine the prevalence of hypertension, the level of acceptability of a PBD, and the associated factors among farmers; and (3) to examine the effectiveness of a community-based nursing program for the management of hypertension using a PBD among farmers.

## Methods

### Study Overview

**Figure 1:**
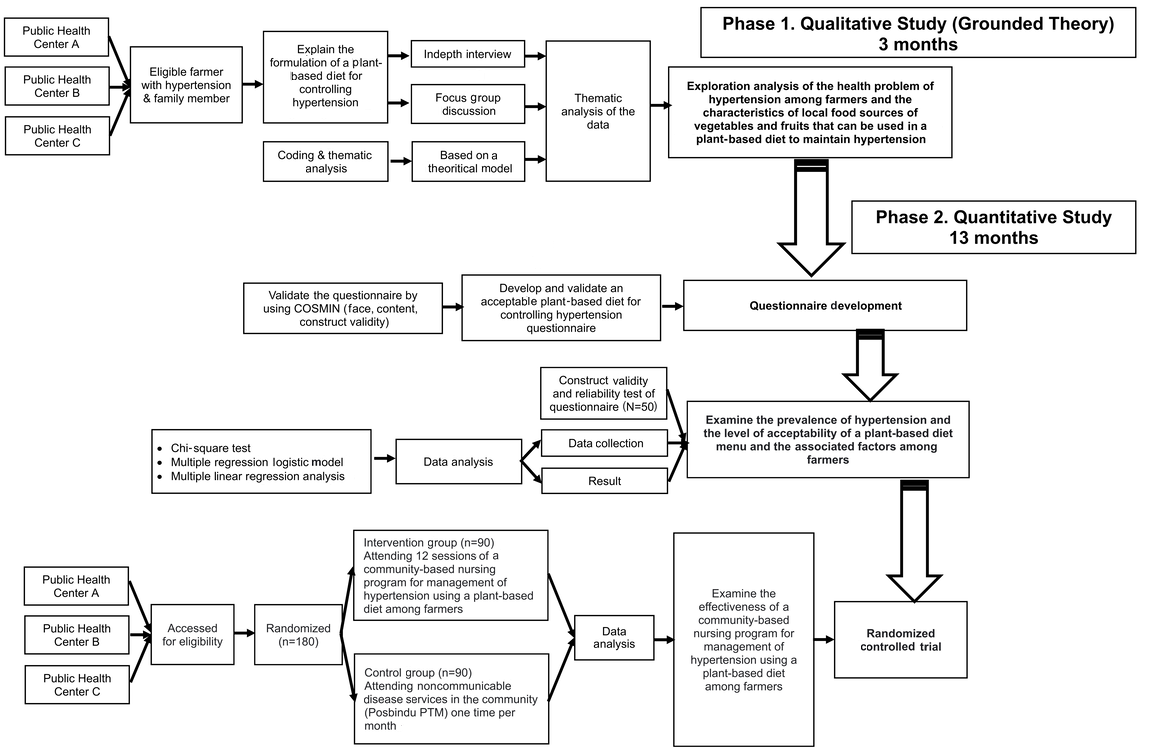
Study overview. COSMIN: consensus-based standards for the selection of health measurement instruments; Posbindu PTM; Pos Pembinaan Terpadu Penyakit Tidak Menular.

The exploratory sequential mixed methods approach will be used to achieve the research objectives in the following 2 phases: qualitative study (phase I) and quantitative study (phase II) (Figure 1). In phase I, we will explore the health problem of hypertension among farmers and the characteristics of local food sources of vegetables and fruits that can be used in a PBD to manage hypertension through semistructured interviews. Then, we will formulate a PBD menu to treat hypertension among farmers.

In phase II, we will conduct a quantitative study. In this phase, the following 3 activities will be performed: (1) we will develop and validate a questionnaire as the assessment instrument; (2) we will examine the prevalence of hypertension, the level of acceptability of a PBD, and the associated sociodemographic factors among farmers; and (3) we will examine the effectiveness of a community-based nursing program for managing hypertension using a PBD among farmers.

This study will use farmers with hypertension and their families in the planning of phases I and II.

### Phase I: Qualitative Phase

#### Overview

We will perform a semistructured interview involving farmers with hypertension and the family members of eligible participants to address the research objectives. The qualitative study will provide insights on health problems related to hypertension and the characteristics of local food, and will help formulate a PBD to control blood pressure from the perspective of agricultural health nursing care services. After undergoing training in conducting qualitative studies, 5 investigators (TS, HR, FK, IR, and RAY) will conduct interviews in the field.

#### Setting

This study will recruit eligible participants selected from 3 public health centers (PHCs) in Jember, East Java Province of Indonesia. Jember, East Java Province is a province representing Indonesia’s agricultural sector. It is known that Jember Regency is an agricultural area, and most of its people make a living as farmers or work in agriculture. The agricultural land in Jember Regency is very fertile. Therefore, most land in Jember Regency is used for agriculture and plantations. This is very suitable as the main livelihood of the people of Jember Regency is related to the agricultural sector.

Jember Regency is considered one of the rice barns of East Java Province, reflecting that the agricultural sector plays an important role (leading sector) in the economy of Jember Regency, and it can be said that Jember has an agrarian economic structure. Since Jember’s economy is generally based on agriculture, the maintenance and management of natural resources are very important, and it is hoped that the economic development carried out is oriented toward environmentally sound development. Regarding cultivations, all areas except the city center have food crops; the slopes of Mount Argopuro have plantations of tea, coffee, cocoa, and rubber; the slopes of Mount Raung have plantations of coffee and tobacco; and central to southern areas have plantations of tobacco, sugarcane, and coconut. In Jember, 42.9% of the population has jobs in agriculture [17]. Based on data [18] released by BPS Kabupaten Jember, the population of Jember Regency in 2020 was projected to reach 2,536,729 people, consisting of 1,264,968 male residents and 1,271,761 female residents, and the population density in Jember Regency in 2020 reached 770.26 people/km^2^.

#### Participants

The inclusion criteria for recruiting the purposive sample are as follows: (1) farmers aged 20-65 years from other parts of East Java Province, Indonesia (citizens) living in 3 PHCs of Jember; (2) residents living in 3 PHCs of Jember; (3) those with hypertension; (4) those who consume fruits and vegetables to control their blood pressure; (5) those who attend the Integrated Health Center (IHC) for noncommunicable diseases (NCDs) in the community; and (6) those having and living with family for care provision. We will exclude older people who are not citizens of Indonesia and those who are unwilling to be interviewed or unable to communicate clearly due to language problems. To obtain more insights on the acceptability of a PBD, the family members of eligible farmers with hypertension will be included in the interview. The inclusion criteria are as follows: (1) employment as a farmer; (2) age 45-65 years; (3) presence of grade I/II hypertension according to the Eighth Joint National Committee on Prevention, Detection, and Treatment of High Blood Pressure (JNC VIII) criteria; and (4) presence of relatives who stay in the same house with their parents or take care of their parents by helping them with preparing food or assisting them in daily routines. We will exclude farmers with hypertension having other chronic diseases (diabetes mellites, chronic kidney disease, heart disease, and stroke).

#### Sampling Process

Phase I is estimated to last 3 months. The snowball sampling method will be used to recruit participants. The investigators (TS, HR, FK, IR, and RAY) will recruit participants in Jember based on the inclusion and exclusion criteria. The interviewees will be asked to recommend other eligible participants among farmers and their family members who wish to participate in this research. Investigators will contact eligible participants to explain the study details, confirm their interest in participating, and determine their preferred interview method (eg, individual interview or focus group discussion). Payment of US $10 will be made to participants for participating in this study (online transfer to their bank accounts).

#### Data Collection

Phase I will involve the following 2 data collection methods: in-depth interviews and focus groups using semistructured interview guides. Further, in-depth interviews may be conducted with certain respondents who have attended the previous focus group interviews to gain more perspectives and clarify the focus groups’ findings. Each focus group will consist of 4 to 6 participants. An earlier scoping review conducted by the same research team will help identify health problems related to hypertension among farmers. Then, we will explore the characteristics of local food sources to manage hypertension and formulate a PBD. The findings will provide additional information for developing the interview guides and questions (eg, “Can you describe your health problem related to your hypertension?” “How would you control your blood pressure with a plant-based diet?” “What kinds and contents of local foods would you use for controlling hypertension?” “How would you formulate a plant-based diet menu to control hypertension?” “What do you expect from a plant-based diet?” and “Would you adopt a plant-based diet? If yes, why? If no, why not?”). The preliminary semistructured interview guides, which contain open-ended questions and probes to explore the acceptability of a PBD, will be pilot tested.

We plan to interview the farmers and the family members of eligible farmers with hypertension. We will recruit 30 participants initially, but the final sample size for this qualitative study will be determined when data saturation is reached, for example, when additional data collection reveals no substantial new information. Each in-depth interview will take between 30 and 45 minutes, and the focus group interview will take around 45 to 60 minutes. Before the interviews, participants will provide their sociodemographic and related information, such as age, education, occupation, income, insurance, and health condition. Information about this research, including the hypertension problem and the characteristics of local foods used in a PBD, will be explained to all participants in Bahasa Indonesia before they are asked to provide informed consent. All interviews will be conducted in the Bahasa Indonesia language and will be audio recorded. Investigators will take down field notes, and transcripts will be translated line by line into English. All data will be translated into English to allow discussions with non-Indonesian researchers.

#### Qualitative Data Analysis Plan

Interviews will be video recorded, and all data will be transcribed verbatim. Data in Bahasa Indonesia will be translated into English for co-investigators who are only proficient in English. Qualitative data will be analyzed to identify a thematic framework [19], guided by a theoretical model of a PBD for controlling hypertension in farmers [20]. The emerging codes will be analyzed thematically according to the framework if they are coherent. The team members will repeatedly read each document and transcript to familiarize themselves with the whole data set and ensure the accuracy of the transcription reviews. Codes will be applied to raw data and then grouped into clusters to generate themes. Consensus on the codes and themes must be reached among at least three of the investigators (TS, HR, FK, IR, and RAY). The data will be compared in an ongoing manner to identify the true meaning of the data. Member checking and investigator reflexivity will be used to increase the credibility and reliability of the data. Data credibility will be enhanced through further analysis of interview and field notes, and views from the different categories of farmers having different sociodemographic backgrounds (eg, those with different grades of hypertension), as well as through triangulation involving multiple investigators. The content of the interviews will be shared with some participants, and their feedback on the findings will be sought for validation purposes (respondent validation). Investigators involved in the data analysis will collect and explain their biases or assumptions during data analysis and interpretation (reflexivity). Raw data, interpretations, and conclusions will be discussed with peers (peer debriefing). Confirmability will be enhanced by keeping an audit trail of the audio/video files, electronic documents, original field notes, and translations [21].

### Phase II: Quantitative Phase

#### Activity 1: Questionnaire Development and Validation

##### Overview

In activity 1, we will develop a questionnaire of an acceptable PBD for controlling hypertension, according to the qualitative data in phase I. The quotations from raw data will become questionnaire items, themes will become scales, and codes will become variables. A Likert score will be used for measurement (range of 1 to 4 points), which will determine the levels of expectations and acceptability. Based on the scores of the items or the summed scores, expectations and acceptability can be categorized into the following 3 categories: affordable, accessible, and achievable. Categorization will be performed using a statistical formula. If the data are normally distributed, the grade level will be based on the mean value; otherwise, the median will be used (X < mean/median – 1 SD; mean/median – 1 SD < X < mean/median + 1 SD; mean/median + 1 SD < X).

The questionnaire will have the following 2 sections: (1) sociodemographic characteristics of hypertension and (2) acceptable PBD for controlling hypertension. The instructions and descriptions of the definition, concept, and criteria of a PBD will be provided at the beginning of the questionnaire. The Pender Health Promotion Model will guide the questionnaire’s qualitative analysis and item design. After performing a member check and assessing face and content validity with an expert committee, we expect the final version of the questionnaire. The expert committee will include investigators with expertise in qualitative research (HR), statistical analysis (FK), public health (TS), and research methodology (RAY and IR).

##### Validation and Reliability

A cognitive debriefing involving 10 people to further assess the face and content validity of the questionnaire will be performed (5 expert nurses on hypertension and 5 nutritionists). Feedback will be discussed by the expert committee, and it will determine the appropriateness and completeness of the questionnaire and ethical considerations in the process. The next analysis step is the COSMIN (consensus-based standards for the selection of health measurement instruments) checklist developed in an international Delphi study to estimate the quality of selected measurement instruments. It measures internal consistency, reliability, measurement error, content validity (as well as face validity), structural validity, hypothesis testing, cross-cultural validity (the 3 aspects of construct validity), criterion validity, and responsiveness. Based on the checklist, items will be sorted by experts in the group to establish face, content, and construct validity in activity 1. The duration to finish the questionnaire is estimated to be less than 30 minutes. Further validation of the questionnaire will be conducted in activity 2. Cronbach alpha will be applied to prove the internal consistency (0.70 or higher is considered as good) of the expectations and acceptability questionnaire. Exploratory factor analysis using principal component analysis will be performed to examine the structural validity of the expectations and acceptability questionnaire separately.

In addition, the Kaiser-Mayer-Olkin test of sampling adequacy, the Bartlett test of sphericity, and the scree plot will be used to check the unidimensionality of the expectations and acceptability questionnaire. The convergence criteria will be set at an eigenvalue above 1. Construct validity (hypothesis testing) will be tested when comparing the responses to the expectations or acceptability of 1 item (yes or no). We hypothesize that the highest tertile expectation will be associated with willingness measured by a repeat measurement value of at least 2.0. The highest tertile acceptability will be related to willingness measured by a repeat measurement value of at least 3.0. It is also hypothesized that expectations and acceptability will correlate at a coefficient of at least 0.4. One-month intrarater test-retest reliability will be assessed in a random sample of at least 50 older people throughout the data collection period in activity 2.

#### Activity 2: Survey

##### Overview

In activity 2, we will conduct a survey to identify the prevalence of hypertension, the level of acceptability of a PBD, and the associated sociodemographic factors among farmers. We will use the questionnaire developed in activity 1. Written informed consent will be obtained, and each participant will be required to indicate agreement for participation before filling out the questionnaire and submitting it.

##### Setting

To obtain a representative sample according to the criteria that have been set, we will select 3 PHCs located in agricultural areas in Jember, East Java, Indonesia. The PHCs we have selected include Banjarsengon, Sukorambi, and Panti. There are quite a lot of farmers in the Panti area, with 14,322 active farmers having quite complex hypertension problems [22]. Farmers in Banjarsengon and Sukorambi also show a similar condition. These 3 PHCs are expected to have a sufficient number of farmers with hypertension who could respond and complete the questionnaire.

##### Participants

The criteria for recruiting our representative sample are as follows: (1) farmers aged 20-65 years from other parts of East Java Province, Indonesia (citizens) living in 3 PHCs in Jember; (2) residents living in 3 PHCs in Jember; (3) those with grade I/II hypertension according to the JNC VIII criteria; (4) those who attend the IHC for NCDs in the community; and (5) those having and living with family for care provision. We will exclude (1) farmers with psychiatric disorders that may impair their ability to answer or complete the questionnaires and (2) farmers with cognitive impairment.

##### Sample Size Estimation

The sample size will be determined based on a G*Power analysis. Since no previous similar study has been conducted, this study takes the best estimate of the minor difference in the proportion of expectations and acceptability of a smart nursing home among elderly people to be at 10%. Using G*Power 3.1.2 (Institute for Experimental Psychology in Dusseldorf Germany) with 0.90 power and significance at a 2-sided α of .05, the estimated sample size is 263.

##### Measures

To meet the study objectives, the questionnaire will consist of the following sections: (1) sociodemographic characteristics and (2) the level of acceptability of a PBD to treat hypertension among farmers. The sociodemographic characteristics will include age, gender, marital status, education (no formal education, elementary school, middle school, and high school or higher), household monthly income per capita (less than US $65.82, US $65.82-131.65, US $131.66-197.47, and over US $197.47), duration of hypertension (years), grade of hypertension based on the JNC VIII criteria, and living condition (with children or a spouse). In section 2 of the questionnaire, a 4-point Likert scale will be used to assess the level of acceptability of a PBD to treat hypertension among farmers. Expectations will be measured as follows: 1, strongly disagree; 2, disagree; 3, agree; and 4, strongly agree. A higher score will indicate a higher level of acceptability of a PBD to treat hypertension among farmers. The association of sociodemographic factors with the different categories of expectations and acceptability will be determined through statistical analysis.

##### Sampling Process

Representative research respondents will be selected by collecting available data on public health services. Farmers with hypertension are listed as regular patients in the Posbindu PTM (Pos Pembinaan Terpadu Penyakit Tidak Menular) program of the public health services. The data obtained will be assessed in a random process to select respondents using a simple random sampling technique. We will use a randomizer application that is available online to select respondents. Respondents selected from the randomizer application will then be included in the list of research respondents. We will follow the activities of the Posbindu PTM program as well as meet with research respondents who have been selected to be able to collect research data. We will print out the questionnaire and give it to the research respondents to fill out. Our team will assist farmers in filling out questionnaires to ensure that the questionnaires given are filled out completely and accurately.

##### Analysis Plan

The data from the questionnaire will be inputted into SPSS (IBM Corp) for analysis. We will perform data cleaning to check each data point from the questionnaire and ensure there are no missing data from the entire questionnaire. We will only use completed questionnaires for analysis. After the data cleaning process is complete, we will perform the data analysis process.

We will use descriptive statistics to describe the characteristics of the respondents and the level of acceptability of a PBD to treat hypertension among farmers according to data distribution. Numerical variables will be shown as means and standard deviations or medians and interquartile ranges, while categorical variables will be shown as absolute frequencies and percentages. We will use the chi-square test to examine the sociodemographic factors associated with farmers’ different categories of acceptability. With the 95% CI, a *P* value of <.05 will be considered statistically significant.

We will use multiple logistic regression models to estimate the associated and independent factors from the sociodemographic factors and the level of acceptability of a PBD among farmers. In the case of a low event rate and low sample size for expectations and acceptability in the definite form, we may recode them into tertiles and use the lowest score category as the reference group. Alternatively, we may conduct multiple linear regression analyses with the 2 dependent variables as continuous variables. We will also include any independent factors with a *P* value <.20 from the univariable regression analysis in the multiple regression analysis. Multicollinearity between independent variables will be checked according to a tolerance value <0.4 (variance inflation factor ≥2.5). In the presence of multicollinearity, we will select the more critical or essential variables from clinical perspectives for use in the final regression analysis. We will check all models, check Q-Q plots for normality, check residual plots for linearity, and check homogeneity assumptions, as well as assess model fitting.

#### Activity 3: Randomized Controlled Trial

##### Overview

In activity 3, we will perform a randomized controlled trial (RCT) on the effectiveness of a community-based program for managing hypertension using a PBD among farmers. We will evaluate the effectiveness of a PBD menu for the outcomes (blood pressure and acceptability of a PBD) of farmers with hypertension.

##### Design

An RCT will be performed for the community-based program in 2 groups (with pretest and posttest) using the CONSORT (Consolidated Standards of Reporting Trials) guidelines. An RCT is preferred to reduce selection bias [23]. Participants in this study will be randomly allocated to either the control or intervention group. The control group will receive a standard health education intervention for managing hypertension, while the intervention group will receive the PBD formula. A single-blind technique will be used so that participants will be unaware if they are in the control or intervention group.

##### Participants

The target population of this study is farmers with hypertension from 3 PHCs in Jember, East Java, Indonesia. Three PHCs have been chosen to represent the characteristics of agricultural areas with a high hypertension problem. The 3 PHCs have implemented the Integrated Health Program (IHP) for NCDs.

##### Inclusion and Exclusion Criteria

The inclusion criteria are as follows: farmers aged 45-65 years with stage I hypertension (systolic blood pressure [SBP]: 140-159 mmHg and diastolic blood pressure [DBP]: 90-99 mmHg) according to the JNC VIII criteria, farmers performing appropriate work, and those providing a commitment to attend the study for 3 months. We will exclude those who have experienced stroke as they are not eligible for participation, those with hypertension of stage II or higher, those taking medications, and those who have been hospitalized.

##### Sample Size Determination

The sample size will be determined using a traditional sample size calculation based on the McNemar test. The sample size in the McNemar test in this study should fulfill the following criteria: (1) directional hypothesis for 1 tail; (2) magnitude of expected change using the odds ratio (OR; OR=2); (3) alpha value of .05; and (4) required power of 0.8 or higher. The OR and the proportion are expected to change as a result of the intervention. Considering a 2-tailed approach, an effect size of 2.0 at an α value of .05 and power of 80%, and a change in 75% of the population as a result of the intervention, 96 participants would be needed to detect the effect.

##### Randomization

Initially, public health nurses (PHNs) will visit the study areas and make announcements about the study for farmer participation. The recruitment will be open for 6 months. Farmers will visit the PHC and will be provided an explanation of the study. Informed consent will be obtained, and farmers will be randomized using a computer-generated number. The allocation sequence will be concealed from the researchers and farmers. The computer-generated number system will randomize the initial name of the farmers from the PHC. The PHNs from the PHC will then obtain informed consent and manage the study groups.

##### Outcome Measures

The main outcome measures in this study are SBP and DBP, which will be measured using a standard protocol employing a stethoscope and sphygmomanometer after stabilizing subjects for more than 10 minutes. Measurements of SBP and DBP (mmHg) will be categorized according to the JNC VIII criteria to determine hypertension for participants aged >18 years [24]. The length of work per day, time of rest during working, and hours of sleep per day will be assessed among the farmers. These factors will be measured as they can influence blood pressure among farmers, according to a previous study [2]. Manual blood pressure measurement is usually performed using a mercury device or a calibrated needle (aneroid). SBP and DBP are determined through auscultation by listening for the appearance and disappearance of Korotkoff sounds in the brachial artery. Blood pressure measurements will be taken after a 14-minute rest period, during which the researcher and the participant will have a conversation to reduce tension associated with the “white coat” effect. Measurements will be taken at the same time (ie, 8 AM before activities) with the same tool. Participants will be told not to wear clothes that are too thick. Blood pressure test results will be more accurate if the cuff is placed directly over the skin. Participants will be told to empty their bladder before measurement. For the measurement, participants will be told to sit with their arms on a table so that the elbows are in line with the heart, the back is well supported by the back of the chair, and the feet are on the floor.

##### Data Collection Plan

At baseline, sociodemographic data, including age, gender, education level, smoking, hours of work, rest during working, and hours of sleep per day, will be obtained using a self-administered questionnaire. For measuring SBP and DBP at the PHC, participants will sit in a chair and relax for 10 minutes. Then, PHNs will measure blood pressure at least twice. If the blood pressure values obtained in the second measurement differ from the values in the first measurement by ≥10 mmHg, a third measurement will be carried out. The 2 measurement values with the smallest difference will be averaged to obtain the final value [24].

##### Intervention Group Arm

The intervention group will receive a community-based intervention program involving a PBD.

##### Control Group Arm

The control group will receive a standard education intervention through the IHC for NCDs and available health services that can be accessed every month in the community.

##### Intervention

The intervention group will receive a PBD formulated by the researchers. Farmers will consume the plant-based food 3 times per week. For the PBD, we will adopt a standard protocol from previous studies [1]. Therefore, we have decided to provide the PBD for 6 months.

In the control group, participants will receive education on health and safety on attending the IHP for NCDs, and they will adopt healthy behaviors according to the health education. As they will receive health education, they will be able to lead a healthy life in the agricultural environment, with occupational health and safety.

In both study groups, health education will be provided before the program, which will include information on how to manage a healthy lifestyle while working as farmers, including diet, exercise, rest, and sleep, and how to address blood pressure problems among farmers. Participants will receive this health education in a single session for 60 minutes at the first screening meeting. All participants will receive a manual book on a healthy lifestyle in an agricultural setting.

The blood pressure of participants in both study groups will be maintained. If the blood pressure increases to hypertension stage II or above, appropriate treatment will be provided by the medical doctor in the PHC and the participant will be excluded from the study. Data will be obtained at registration and at the pretest and posttest assessments. For blood pressure data, we will use rounded values, with values ending in 0-4 being rounded to “0” and values ending in 6-10 being rounded to “10” (eg, 120-124 mmHg will be rounded to 120 mmHg, 125 mmHg will be used directly, and 126-130 mmHg will be rounded to 130 mmHg).

##### Data Analysis Plan

SPSS version 22.0 (IBM Corp) will be used for the analysis. The significance level will be set as *P*<.05. Numerical data will be presented using means and standard deviations, while categorical data will be presented using frequencies and percentages. Furthermore, a linear generalized estimating equation (GEE) will be used to estimate the parameters of a generalized linear model with a possible unmeasured correlation between observations from different timepoints for SBP and DBP (comparing pretest and posttest).

### Ethical Considerations

This study has received ethical approval from the Ethics Committee for Research Involving Human Subjects, Faculty of Dentistry of Universitas Jember (approval number: 1548/UN25.8/KEPK/DL/2022, with an amendment on June 29, 2021) and conforms to the requirements of the World Medical Association’s Declaration of Helsinki. All the participants will provide written informed consent before their inclusion.

This study will use descriptive analytics in health and community service units with a quantitative approach through surveys and qualitative interviews at health and community service units. This research will not cause harm to the participants. Each participant will provide informed consent. We will give autonomy rights to prospective participants. Participants will be provided an explanation of the study, including the objectives, benefits, implementation process, and participant rights. Participants will be informed that there is no risk of harm if they do not participate in the study or leave the study while the research process is in progress.

The study will pay attention to safety aspects for both the researchers and participants, and will be performed according to the applicable COVID-19 protocol. The interviews will be adjusted according to the participants’ time. Participants will receive compensation (transportation fee) and food for each data collection. The design of this study involves an intervention group and a control group, and we will try to minimize the occurrence of unwanted incidents during the implementation of the intervention. We will provide leaflets containing educational materials for a PBD when the last measurement is taken. Participants in the control group will be offered a discussion session after reading the leaflet provided, but there is no compulsion to join the discussion session. All participants in the intervention group will receive the same intervention regardless of ethnicity, religion, and race.

The confidentiality of the participants’ data will be maintained, and the identity of the participants will not be disclosed. The data obtained will only be used for research purposes. To keep participants’ data confidential, the survey sheet will have a special code that does not display the personal data. Moreover, a statement will be provided that qualitative study data are anonymous or deidentified. Furthermore, a brief description of the protections in place will be provided.

## Results

This research has been funded by DRPTM Kementerian Pendidikan, Kebudayaan, Riset dan Teknologi Skema Riset Penelitian Dasar Unggulan Perguruan Tinggi (PDUPT) in 2022 (0277/E5/AK.04/2022). The research started in May 2022, and phase I of the study is scheduled to begin in May 2023, with a total research duration of 2 years. A model PBD for the management of hypertension is expected to be developed from using local food–related agronursing services. In 2022, we will collect data on hypertension and the characteristics of local food sources to manage hypertension, and formulate a PBD menu to treat hypertension among farmers. A thematic analysis of the qualitative data of farmers in the agricultural sector will be performed. In 2023, we will develop a questionnaire to assess the acceptability of a PBD to manage hypertension among farmers, the prevalence of hypertension, and the sociodemographic factors associated with hypertension among farmers. Furthermore, we will introduce a community-based nursing program for the management of hypertension using a PBD among farmers.

## Discussion

### Contributions of the Study

This paper describes a protocol for the management of hypertension using a PBD among farmers. We hypothesize that the formulation of the PBD concept can overcome the problem of hypertension among farmers in agricultural areas, which is synergistic with the culture and local wisdom of the agricultural population. The findings of this study may contribute to the development of a better solution for hypertension among farmers in Indonesia and may be relevant to a special group of readers interested in this research. It is believed that innovations of the intervention can be used and integrated to promote nursing care within the fields of health assessment, activities of daily living, and care management, which could improve the quality of life and quality of care for farmers in agricultural areas.

The concept of a PBD as a solution for hypertension is based on meeting the nutritional needs of plant-based proteins and minimizing intake from animal and processed sources [24]. Maximized intake of nutrients in a PBD involves high intake of fiber, vegetable protein, vitamins, polyphenols, and unsaturated fatty acids, and low intake of sodium. Food processing in a PBD can be adapted to the culture and habits of the local community to facilitate the consumption process and maintain consistency in the implementation of the process. A PBD is expected to provide considerable benefits to overcome hypertension in farmers. Optimal nutrition and fiber in a PBD will provide strong protection for organ systems in the body and will help systems to work synergistically to reduce inflammation and oxidation in the body [19].

A PBD model will provide farmers with an alternative solution that has independent, safe, and comfortable features for their needs. It could also be used to adapt and transform existing hypertension programs for farmers and their family members. With a better understanding of farmers’ expectations and acceptability of a PBD model in public health, researchers, stakeholders, and policymakers may be able to effectively develop a complete model that incorporates appropriate technologies and integrates relevant medical services, and yet remains affordable to benefit the majority of farmers. This would facilitate the transformation of traditional hypertension interventions to smart and local interventions, which include necessary skill sets or required training for PHNs and other health care professionals to meet expectations.

The findings of this study will be presented at conferences and published in peer-reviewed journals. We will publish the results of this research in academic journals. We will also present this research at national and international conferences. The data from this study will be presented in a research report to the Ministry of Cultural Education, Research and Technology, and Higher Education on the BIMA system dashboard (BIMA – Kemdikbudristek) as restricted data. In addition, research data will be reported to the Institute for Research and Community Service, University of Jember (organizer in research activities). Furthermore, the research results of the first year and second year will be presented in a manuscript. The manuscript will be submitted to a reputable international journal in the areas of hypertension and NCD management.

Disseminating our study findings will significantly contribute to the advancement of science and holistic nursing services for farmers with hypertension in the community.

### Limitations of the Study

This study will examine the effectiveness of a community-based nursing program for the management of hypertension using a PBD among farmers in Indonesia. However, this protocol may have some limitations. First, the program requires participant compliance for carrying out regular training to maintain blood pleasure, and some participants may drop out of this study for various reasons, such as noncompliance with the consumption of a PBD. Therefore, a dairy is required to continuously monitor the diet. Second, the measurement of blood pressure may be biased because of different enumerators and sphygmomanometers. This can be minimized by interrater enumerator measurements and instrument calibration.

### Conclusion

We believe the findings of this research will be beneficial for farmers in the agricultural and plantation areas of Jember, who experience hypertension. We will compile the results of our research by comparing and discussing the results in terms of the results of previous similar studies. Future research is expected to improve the results of this study in terms of determining the preparation of hypertension management using a PBD for farmers. We expect contributions from the local government to implement the intervention as one of the policies in the management of hypertension for farmers in the agricultural plantation areas of ​​Jember. This program may also be implemented in other agricultural countries with similar problems, so that hypertension can be optimally treated among farmers.

## Abbreviations

DBP: diastolic blood pressure
IHC: Integrated Health Center
IHP: Integrated Health Program
JNC VIII: Eighth Joint National Committee on Prevention, Detection, and Treatment of High Blood Pressure
NCD: noncommunicable disease
OR: odds ratio
PBD: plant-based diet
PHC: public health center
PHN: public health nurse
RCT: randomized controlled trial
SBP: systolic blood pressure
